# Derivation and validation of four patient clusters in Still’s disease, results from GIRRCS AOSD-study group and AIDA Network Still Disease Registry

**DOI:** 10.1136/rmdopen-2023-003419

**Published:** 2023-11-20

**Authors:** Piero Ruscitti, Francesco Masedu, Antonio Vitale, Ilenia Di Cola, Valeria Caggiano, Claudia Di Muzio, Paola Cipriani, Marco Valenti, Onorina Berardicurti, Luca Navarini, Daniela Iacono, Ilenia Pantano, Daniele Mauro, Francesco Ciccia, Silvia Rossi, Ludovico De Stefano, Sara Monti, Serena Bugatti, Carlomaurizio Montecucco, Francesco Caso, Luisa Costa, Marcella Prete, Federico Perosa, Annamaria Iagnocco, Fabiola Atzeni, Giuliana Guggino, Henrique Giardini, Isabele Parente de Brito Antonelli, Ibrahim A Almaghlouth, Kazi Asfina, Haner Direskeneli, Fatma Alibaz-Oner, Gizem Sevik, Abdurrahman Tufan, Petros P Sfikakis, Francesco La Torre, Andrea Hinojosa-Azaola, Eduardo Martín-Nares, Jiram Torres-Ruiz, Gafaar Ragab, Maria Cristina Maggio, Joanna Makowska, Emanuela Del Giudice, Elena Bartoloni, Giacomo Emmi, Marcello Govoni, Alberto Lo Gullo, Giuseppe Lopalco, Gabriele Simonini, Lampros Fotis, Benson Ogunjimi, Samar Tharwat, Bruno Frediani, Armin Maier, Francesco Carubbi, Lorenzo Dagna, Sukran Erten, Antonio Gidaro, José Hernández-Rodríguez, Paolo Sfriso, Claudia Fabiani, Roberto Giacomelli, Luca Cantarini

**Affiliations:** 1 Rheumatology Unit, Department of Biotechnological and Applied Clinical Sciences, University of L'Aquila, L'Aquila, Italy; 2 UOC Reumatologia, Azienda Ospedaliero-Universitaria Senese, ERN-RITA Center, Siena, Italy; 3 Department of Medical Sciences, Surgery and Neurosciences, Research Center of Systemic Autoinflammatory Diseases and Behçet's Disease Clinic University of Siena, Siena, Italy; 4 Rheumatology and Clinical Immunology, Department of Medicine, University of Rome “Campus Biomedico”, School of Medicine, Rome, Italy; 5 Clinical and Research Section of Rheumatology and Clinical Immunology, Fondazione Policlinico Campus Bio-Medico, Rome, Italy; 6 Department of Precision Medicine, University of Campania "Luigi Vanvitelli", Naples, Italy; 7 Rheumatology Department, Istituto di ricovero e cura a carattere scientifico Policlinico S. Matteo Fondazione, University of Pavia, Pavia, Italy; 8 Rheumatology Unit, Department of Clinical Medicine and Surgery, University of Naples Federico II, Naples, Italy; 9 Rheumatic and Systemic Autoimmune Diseases Unit, Department of Interdisciplinary Medicine (DIM), University of Bari Medical School, Bari, Italy; 10 Academic Rheumatology Centre, Ospedale Mauriziano - Dipartimento Scienze Cliniche e Biologiche, Università degli Studi di Torino, Turin, Italy; 11 Rheumatology Unit, Department of Clinical and Experimental Medicine, University of Messina, Messina, Italy; 12 Rheumatology Section, Department of Health Promotion, Mother and Child Care, Internal Medicine and Medical Specialties, University Hospital P. Giaccone, Palermo, Italy; 13 Rheumatology Division, Faculdade de Medicina, Hospital das Clínicas, Universidade de São Paulo, São Paulo, Brazil; 14 Rheumatology Unit, Department of Medicine, College of Medicine, King Saud University, Riyadh, Saudi Arabia; 15 College of Medicine Research Center, College of Medicine, King Saud University, Riyadh, Saudi Arabia; 16 Department of Internal Medicine, Division of Rheumatology, Marmara University, School of Medicine, Istanbul, Turkey; 17 Division of Rheumatology, Department of Internal Medicine, Gazi University Faculty of Medicine, Ankara, Turkey; 18 Joint Academic Rheumatology Program, National and Kapodistrian University of Athens Medical School, Athens, Greece; 19 Clinical Pediatrics, University of Bari, Bari, Italy; 20 Department of Immunology and Rheumatology, Instituto Nacional de Ciencias Médicas Y Nutrición Salvador Zubirán, Mexico City, Mexico; 21 Internal Medicine Department, Rheumatology and Clinical Immunology Unit, Faculty of Medicine, Cairo University, Giza, Egypt; 22 Faculty of Medicine, Newgiza University, 6th of October City, Egypt; 23 University Department Pro.Sa.M.I. "G. D'Alessandro", University of Palermo, Palermo, Italy; 24 Department of Rheumatology, Medical University of Lodz, Łódź, Poland; 25 Pediatric and Neonatology Unit, Department of Maternal Infantile and Urological Sciences, Sapienza, University of Rome, Polo Pontino, Latina, Italy; 26 Department of Medicine and Surgery, MED/16- Rheumatology, Università degli studi di Perugia, P.zza Università, Perugia, Italy; 27 Department of Experimental and Clinical Medicine, University of Florence, Florence, Italy; 28 Centre for Inflammatory Diseases, Monash University Department of Medicine, Monash Medical Centre, Melbourne, Victoria, Australia; 29 Rheumatology Unit, Department of Medical Sciences, Azienda Ospedaliero-Universitaria S. Anna - Ferrara, University of Ferrara, Ferrara, Italy; 30 UOSD Reumatologia ARNAS Garibaldi, Catania, Italy; 31 Rheumatology Unit, Department of Emergency and Organ Transplantation, University of Bari, Bari, Italy; 32 NEUROFARBA Department, Rheumatology Unit, Meyer Children's University Hospital, University of Florence, Firenze, Italy; 33 Third Department of Paediatrics, National and Kapodistrian University of Athens, General University Hospital "Attikon", Athens, Greece; 34 Antwerp Unit for Data Analysis and Computation in Immunology and Sequencing, University of Antwerp, Antwerp, Belgium; 35 Antwerp Center for Translational Immunology and Virology, Vaccine and Infectious Disease Institute, University of Antwerp, Antwerp, Belgium; 36 Department of Paediatrics, Antwerp University Hospital, Antwerp, Belgium; 37 Center for Health Economics Research and Modeling Infectious Diseases, Vaccine and Infectious Disease Institute, University of Antwerp, Antwerp, Belgium; 38 Rheumatology and Immunology Unit, Internal Medicine Department, Mansoura University, Mansoura, Egypt; 39 Department of Internal Medicine, Faculty of Medicine, Horus University, New Damietta, Egypt; 40 Rheumatology Unit, Department of Medicine, Central Hospital of Bolzano, Bolzano, Italy; 41 Department of Life, Health & Environmental Sciences and Internal Medicine and Nephrology Unit, Department of Medicine, University of L'Aquila and ASL Avezzano-Sulmona-L'Aquila, San Salvatore Hospital, L'Aquila, Italy; 42 Unit of Immunology, Rheumatology, Allergy and Rare Diseases, IRCCS San Raffaele Hospital, Milan, Italy; 43 Vita-Salute San Raffaele University, Milan, Italy; 44 Department of Rheumatology, Faculty of Medicine Ankara City Hospital, Ankara Yıldırım Beyazıt University, Ankara, Turkey; 45 Department of Biomedical and Clinical Sciences Luigi Sacco, Luigi Sacco Hospital, University of Milan, Milan, Italy; 46 Vasculitis Research Unit and Autoinflammatory Diseases Clinical Unit, Department of Autoimmune Diseases, Hospital Clinic of Barcelona, IDIBAPS, University of Barcelona, [European Reference Network (ERN) for Rare Immunodeficiency, Autoinflammatory and Autoimmune Diseases (RITA) Center], Barcelona, Spain; 47 Rheumatology Unit, Department of Medicine (DIMED), University of Padova, Padova, Italy; 48 Ophthalmology Unit, Department of Medicine, Surgery and Neurosciences, University of Siena and Azienda Ospedaliero-Universitaria Senese [European Reference Network (ERN) for Rare Immunodeficiency, Autoinflammatory and Autoimmune Diseases (RITA) Center], Siena, Italy

**Keywords:** Still's Disease, Adult-Onset, Arthritis, Juvenile, Arthritis

## Abstract

**Background:**

Different patient clusters were preliminarily suggested to dissect the clinical heterogeneity in Still’s disease. Thus, we aimed at deriving and validating disease clusters in a multicentre, observational, prospective study to stratify these patients.

**Methods:**

Patients included in GIRRCS AOSD-study group and AIDA Network Still Disease Registry were assessed if variables for cluster analysis were available (age, systemic score, erythrocyte sedimentation rate (ESR), C reactive protein (CRP) and ferritin). K-means algorithm with Euclidean metric and Elbow plot were used to derive an adequate number of clusters.

**Results:**

K-means clustering assessment provided four clusters based on means standardised according to z-scores on 349 patients. All clusters mainly presented fever, skin rash and joint involvement. Cluster 1 was composed by 115 patients distinguished by lower values of age and characterised by skin rash myalgia, sore throat and splenomegaly. Cluster 2 included 128 patients identified by lower levels of ESR, ferritin and systemic score; multiorgan manifestations were less frequently observed. Cluster 3 comprised 31 patients categorised by higher levels of CRP and ferritin, they were characterised by fever and joint involvement. Cluster 4 contained 75 patients derived by higher values of age and systemic score. Myalgia, sore throat, liver involvement and life-threatening complications, leading to a high mortality rate, were observed in these patients.

**Conclusions:**

Four patient clusters in Still’s disease may be recognised by a multidimensional characterisation (‘Juvenile/Transitional’, ‘Uncomplicated’, ‘Hyperferritinemic’ and ‘Catastrophic’). Of interest, cluster 4 was burdened by an increased rate of life-threatening complications and mortality, suggesting a more severe patient group.

**WHAT IS ALREADY KNOWN ON THIS TOPIC**

- Still’s disease is highly heterogeneous disease and different patient clusters were proposed according to age of onset, diverse clinical manifestations and presence of life-threatening complications.

**WHAT THIS STUDY ADDS**

- Four distinct patient clusters in Still’s disease were derived and validated based on a clinical and laboratory multidimensional characterisation (ie, ‘Juvenile/Transitional’, ‘Uncomplicated’, ‘Hyperferritinemic’ and ‘Catastrophic’).
- Each one of these clusters showed some different clinical features from others accounting for the patient heterogeneity in the context of disease continuum.
- Cluster 4 was burdened by an increased rate of multiorgan manifestations and mortality, proposing a more severe patient group to be managed.

**HOW THIS STUDY MIGHT AFFECT RESEARCH, PRACTICE OR POLICY**

- Our findings may suggest that clinical picture-based stratification may be a robust and clinically meaningful approach, addressing the heterogeneity of Still’s disease and tailoring the management on patient characteristics.

## Introduction

Still’s disease is a rare disorder characterised by the typical triad of daily fever, arthritis and skin rash affecting both children and adults.^1 2^ It is codified as a multigenic autoinflammatory disease, at the crossroad of autoinflammatory and autoimmune diseases.^3 4^ Formerly, Still’s disease was named systemic juvenile idiopathic arthritis in children and adult-onset Still’s disease in adults. However, the similarities between paediatric and adult forms have been increasingly recognised.^5 6^ In addition to main manifestations, paediatric and adult patients share other clinical features and laboratory abnormalities.^7 8^ Moreover, the clinical picture of patients with Still’s disease may be burdened by the occurrence of life-threatening complications, mainly macrophage activation syndrome (MAS), a secondary form of haemophagocytic lymphohistiocytosis.^9 10^ Finally, concerning the treatment, diverse immunosuppressive therapies are administered to patients with Still’s disease to target the inflammatory signs and symptoms.^11 12^

Analysing the disease courses of these patients, different patterns are usually recognised: (1) monocyclic, patients with a single episode of the disease; (2) polycyclic, patients characterised by phases of flares alternating with remissions and (3) chronic, patients with a persistent active disease, usually with polyarthritis.^13 14^ However, this classification is not directly based on the clinical features, therefore providing limited information about the management of the disease. In fact, despite a similar clinical picture at the beginning, these patients may have a highly heterogeneous disease according to different manifestations, presence of life-threatening complications and outcomes over time.^15^ In this context, first, a clinical dichotomy was suggested between the two phenotypes of patients with Still’s disease.^16^ The systemic pattern was proposed manifesting with high fever, skin rash and organ damage and the chronic articular pattern showing prominently polyarthritis.^16 17^ However, many patients do not fit neatly into these patterns and no consensus criteria exist for such stratification.^15^ More recently, the application of data mining techniques by using clinical features has been proposed as a further promising strategy for understanding disease heterogeneity and for determining more appropriate therapeutic strategies.^18^ In the context of Still’s disease, different patient clusters were proposed according to age of onset, diverse clinical manifestations and presence of life-threatening complications.^19–21^ Furthermore, to dissect the clinical heterogeneity of Still’s disease, patient manifestations at the beginning were combined with diverse outcomes over time.^22^ By principal component analysis, four different clusters were identified; each one of these showed a prominent different clinical feature from others.^22^ However, a deeper level of clinical categorisation and validation of these patient clusters in Still’s disease are not fully elucidated yet.

On these bases, we aimed at deriving and validating different patient clusters to more accurately dissect the clinical heterogeneity in patients with Still’s disease collected worldwide by two independent study groups, the GIRRCS AOSD-study group and the AIDA Network Still Disease Registry. Furthermore, we aimed at evaluating the different prognostic impacts of derived clusters in these large cohorts of patients with Still’s disease in a multicentre, observational, prospective study.

## Methods

### Patients, settings and study design

Patients included in GIRRCS AOSD-study group and AIDA Network Still Disease Registry were selected if clinical specific variables for cluster analysis were available (ie, age, systemic score, erythrocyte sedimentation rate (ESR), C reactive protein (CRP) and ferritin) and an adequate prospective follow-up to identify a different disease pattern (see below). GIRRCS (*Gruppo Italiano Di Ricerca in Reumatologia Clinica e Sperimentale*) AOSD-study group cohort is a national multicentre study involving Rheumatologic Units throughout Italy, all characterised by high experience in management of Still’s disease as well as in observational studies.^9 22^ Furthermore, patients with Still’s disease were selected among those included in AIDA Network Still Disease Registry, an international, clinical, physician-driven, non-population and electronic-based registry.^23^ Subsequently, a multicentre, observational, prospective study was built considering those patients who were assessed in both cohorts, after the publication of the preliminary derivation study of disease clusters.^22^ For centres included in both study groups, patients were considered once avoiding duplicates. Adult patients fulfilled Yamaguchi criteria and/or Fautrel criteria and/or Cush criteria.^13 24 25^ Paediatric patients fulfilled International League of Associations for Rheumatology (ILAR) criteria for sJIA and/or Paediatric Rheumatology INternational Trials Organisation (PRINTO) provisional criteria for sJIA.^26 27^ The clinical variables of these patients were combined into a cluster analysis to devise possible deeper levels of categorisation of the disease than what previously performed.^22^ After that, a characterisation of the derived clusters was exploited to evaluate possible differences in clinical features, life-threatening complications and disease outcome.

The Ethics Committees of *ASL1 Avezzano-Sulmona-L’Aquila*, L’Aquila, Italy, (Ref. N. 0139815/16; 0095184/20) and of *Azienda Ospedaliero-Universitaria Senese*, Siena, Italy (Ref. N. 14951; NCT05200715) approved the study, which was performed according to the Good Clinical Practice guidelines and the latest Declaration of Helsinki. Written informed consents for involved patients were collected. Clinical data are kept in accordance with the EU General Data Protection Regulations (GDPR), or other counterparts, on the processing of personal data and the protection of privacy (2016/679/EU).

In reporting the results, we followed the Strengthening the Reporting of Observational Studies in Epidemiology (STROBE) guidelines.

### Clinical variables to be addressed

Clinical features, systemic score, life-threatening complications, laboratory markers, therapies and patterns of the disease, were registered.^28 29^ The presence of the following clinical features, at the time of diagnosis, were recorded: fever, typical skin rash, arthralgia or arthritis, myalgia, lymphadenopathy, sore throat, splenomegaly, hepatomegaly or abnormal liver function tests, sore throat and abdominal pain. The diagnosis of pleural effusion or pleuritis, and lung parenchymal involvement was performed by a chest radiograph or CT scan. In patients with the suspicion of lung disease, chest CT scan was performed, and findings codified according to available literature in different main patterns of involvement: (1) multilobar, predominantly peripheral septal thickening, parahilar and/or anterior upper lobes with or without adjacent ground glass opacities; (2) crazy-paving; (3) peripheral consolidations; (4) peribronchovascular consolidations and (5) predominantly ground-glass opacities.^10 30 31^ After clinical examination and chest radiographs, patients with clinical suspicion of pericarditis underwent echocardiography. Taking these features together, each patient was also assessed for the systemic score.^28^ In addition, at the time of diagnosis and during the subsequent follow-up, each patient was evaluated for the presence of life-threatening complications (MAS, thrombotic thrombocytopenic purpura, thrombotic microangiopathy, disseminated intravascular coagulopathy, respiratory distress syndrome, diffuse alveolar haemorrhage, pulmonary arterial hypertension, myocarditis, tamponade, constrictive pericarditis, endocarditis, shock, multiple organ failure, fulminant hepatitis and amyloidosis), as suggested by available literature.^28^ Specifically, the diagnosis of MAS was established according to the available diagnostic criteria.^29^

Furthermore, ESR, CRP and ferritin were recorded at the time of diagnosis. In all patients, other inflammatory diseases, malignancies and infections were ruled out, as previously performed.^28 31^ The administration of therapies, glucocorticoids (GCs), conventional synthetic disease-modifying antirheumatic drugs (csDMARDs) and biological DMARDs (bDMARDs), was also registered and categorised, at the time of diagnosis and during the follow-up based on medications administered to each patient for the longest time-period, as previously reported.^28 32^ In the subsequent follow-up, according to the disease course, patients were categorised into four groups: three clinical patterns (monocyclic, polycyclic and chronic) and death, whichever the course.^13 28^ Specifically, after the diagnosis, there was a fixed follow-up visit at least after 12 months to codify the disease pattern. A monocyclic pattern was defined as a single episode for >2 months but <1 year, followed by sustained remission through the whole follow-up. Remission was defined as the complete disappearance of systemic symptoms and normalisation of laboratory evidence of disease activity for at least two consecutive months, regardless of therapy, as previously performed.^28^ A polycyclic pattern was codified by recurrent systemic flares with remission between flares. A chronic pattern was defined as ≥1 episode of persistent symptoms lasting >1 year. Patients, who died during follow-up, were placed in the fourth group, which was categorised as death associated with Still’s disease or its complications. Thus, all registered deaths were related to Still’s disease.

### Data sources, bias and sample size

Relevant clinical data were collected during the scheduled visits for each involved patient by an extensive clinical history. The Research Electronic Data Capture (REDCap) tool was used to collect and store clinical findings. Considering the observational design, this study could be subjected to a number of possible biases. The main methodological problems were minimised by a careful definition of each variable to be assessed. Furthermore, patients with missing data, which were considered to be meaningful for the clusters analysis, were removed.

A sample size was estimated to assess the probability of correctly detecting that different clusters could be present in our cohort of patients with Still’s disease. According to proposed formula (70*5 variables to include in clusters analysis),^33^ a sample size of 350 patients was adequately calculated to establish how many clusters could be present within the data, and to what extent the cluster membership of individual observations could be accurately classified.

### Statistical analysis

A cluster analysis was performed in order to devise possible deeper levels of categorisation within the data. The k-means algorithm with Euclidean metric was performed, setting 100 random assignments to the cluster seeds. This procedure prevented possible dependence of clusters from the choice of points at the onset. Z-scores were also provided to account for the different units of the selected variables. This methodological choice was performed to minimise the possible confounding effects of different variables units. The Elbow plot was used to devise an adequate number of clusters, avoiding too large choice which could underwent to overfitting. Besides, the cluster plot enabled the view of the separation among clusters using the first two principal components. Accordingly, the clusters display reasonable between and within variability property, which were likely to be not affected by some outliers. Afterwards, the univariate χ^2^ association analysis carried out between clusters and other clinical variables confirmed the consistency of the derived clusters. A multinomial predictive model of the overall outcomes of patients, adjusted by clinical variables, supported the predictive strength of the clusters providing the associated ORs-like. The overall model was tested using the likelihood ratio test. The statistical analysis was performed by using the statistical software R V.4.2.2 (Copyright (C) 2022 The R Foundation for Statistical Computing).

## Results

### Four clusters were derived within this cohort of patients with Still’s disease

Out of patients included in GIRRCS AOSD-study group and AIDA Network Still Disease Registry, 349 patients were assessed because of clinical selected variables for cluster analysis were available (GIRRCS, n=185 patients, AIDA, n=164 patients), as reported in table 1. Among included patients, 55 had a paediatric onset of the disease. In this cohort, 162 patients were treated with bDMARDs, 69.1% with IL-1 inhibitors, 24.7% with IL-6 inhibitor, and 6.2% with TNF inhibitors, respectively.

**Table 1:** Clinical variables of assessed patients with Still’s disease

Clinical characteristics	Cluster 1 ‘Juvenile/transitional’	Cluster 2 ‘Uncomplicated’	Cluster 3 ’Hyperferritinemic’	Cluster 4 ‘Catastrophic’	Coefficient, p value
Number of patients	115	128	31	75	
Clinical variables for clusters analysis					
Age, years, mean±SD	25.4±12.7	39.8±15.2	36.9±16.9	51.8±16.1	**0.30, <0.0001**
Systemic score, mean±SD	6.1±1.7	4.6±1.4	5.5±1.9	6.8±2.0	**0.21, <0.0001**
ESR, mm/hour, mean±SD	96.9±23.4	45.5±21.7	70.2±30.3	93.6±23.6	**0.50, <0.0001**
CRP, mg/L, mean±SD	36.1±37.8	46.3±43.5	146.6±101.1	143.8±103.6	**0.35, <0.0001**
Ferritin, ng/mL, mean±SD	2171.4±2279.6	1581.3±2253.9	17245.4±5328.1	2947.7±2818.6	**0.71, <0.0001**
*Other clinical features*					
Male sex, n (%)	46 (40.0)	63 (49.2)	10 (32.3)	41 (54.7)	6.83, 0.078
Fever, n (%)	111 (96.5)	124 (96.9)	30 (96.8)	74 (98.7)	1.73, 0.631
Joint involvement, n (%)	103 (89.6)	117 (91.4)	27 (87.1)	66 (88.0)	0.87, 0.834
Arthralgia, n (%)	101 (87.8)	113 (88.3)	26 (83.9)	60 (80.0)	3.22, 0.359
Arthritis, n (%)	71 (61.7)	83 (64.8)	17 (54.8)	46 (61.3)	1.13, 0.770
Skin rash, n (%)	83 (72.2)	77 (60.2)	21 (67.7)	55 (73.3)	5.36, 0.147
Sore throat, n (%)	70 (60.9)	61 (47.7)	18 (58.1)	54 (72.0)	**12.25, 0.007**
Myalgia, n (%)	75 (65.2)	65 (50.8)	17 (54.8)	52 (69.3)	**9.19, 0.027**
Lymph node involvement, n (%)	62 (53.9)	51 (39.8)	14 (45.2)	48 (64.0)	**11.99, 0.007**
Liver involvement, n (%)	58 (50.4)	52 (40.6)	18 (58.1)	53 (70.7)	**18.20, <0.0001**
Spleen involvement, n (%)	57 (49.6)	44 (34.4)	11 (35.5)	45 (60.0)	**13.73, 0.003**
Pericarditis, n (%)	18 (15.7)	15 (11.7)	7 (22.6)	23 (30.7)	**12.57, 0.006**
Pleuritis, n (%)	16 (13.9)	14 (10.9)	6 (19.4)	26 (34.7)	**19.61, <0.0001**
Abdominal pain, n (%)	21 (18.3)	14 (10.9)	3 (9.7)	12 (16.0)	3.14, 0.371
Life-threatening complications					
MAS, n (%)	21 (18.3)	9 (7.0)	7 (22.6)	17 (22.7)	**11.82, 0.008**
Lung disease, n (%)	9 (7.8)	5 (3.9)	2 (6.5)	16 (21.3)	**17.98, <0.0001**
Therapies					
GCs, n (%)	109 (94.8)	122 (95.3)	29 (93.5)	71 (94.7)	1.29, 0.730
Low dose GCs, n (%)	46 (42.2)	64 (52.5)	11 (37.9)	28 (39.4)	5.23, 0.156
csDMARDs, n (%)	68 (59.1)	85 (66.4)	22 (71.0)	51 (68.0)	1.79, 0.617
bDMARDs, n (%)	62 (53.9)	57 (44.5)	13 (41.9)	30 (40.0)	6.76, 0.344
Disease courses and time of observation					
Monocyclic, n (%)	49 (42.6)	43 (33.6)	18 (58.1)	20 (26.7)	**20.55, 0.015**
Polycyclic, n (%)	37 (32.2)	46 (35.9)	9 (29.0)	30 (40.0)	
Chronic, n (%)	27 (23.5)	31 (24.2)	4 (12.9)	16 (21.3)	
Mortality, n (%)	2 (1.7)	8 (6.25)	0 (0.0)	9 (12.0)	
Follow-up, years, mean±SD	2.57±3.55	3.35±3.42	2.35±2.77	3.44±3.33	0.01, 0.18

Bold values are statistically significant results.

bDMARDs, biological disease-modifying antirheumatic drugs; CRP, C reactive protein; csDMARDs, conventional synthetic disease-modifying antirheumatic drugs; ESR, erythrocyte sedimentation rate; GCs, glucocorticoids; MAS, macrophage activation syndrome.

By combining clinical selected variables (ie, age, systemic score, ESR, CRP and ferritin), the K-means clustering assessment provided four disease clusters based on means standardised according to z-scores and elbow plot on 349 patients (online supplemental table 1 and online supplemental figure 1). The ‘within’ and ‘between’ separation properties were also derived. Within cluster sum of squares (SS) by cluster was estimated: (1) cluster 1: 251.69; (2) cluster 2: 283.22; (3) cluster 3: 159.98 and (4) cluster 4: 323.76. SS_between_/SS_total_ was derived to be 41.5%. The latter is not particularly high, but not affected by overfitting phenomena. In fact, the derived clusters showed reasonable ‘within’ and ‘between’ variability properties. After that, randomly sampling 50% of the original records, the same number of clusters was provided by the elbow plot with a similar SS_between_/SS_total_ of 42.6%.

10.1136/rmdopen-2023-003419.supp1 Supplementary data

Descriptively, cluster 1 was composed by 115 patients (age: 25.4±12.7 years; systemic score: 6.1±1.7; ESR: 96.9±23.4 mm/hour; CRP: 36.1±37.8 mg/L; ferritin: 2171.4±2279.6 ng/mL). This group of patients was characterised by lower values of age and CRP. Almost all paediatric patients were included in this cluster (49 out of 55).

Cluster 2 included 128 patients (age: 39.8±15.2 years; systemic score: 4.6±1.4; ESR: 45.5±21.7; CRP: 46.3±43.5 mg/L; ferritin 1581.3±2253.9 ng/mL). Lower levels of systemic score and ESR were observed in these patients. Furthermore, cluster 3 comprised 31 (age: 36.9±16.9 years; systemic score: 5.5±1.9; ESR: 70.2±30.3; CRP: 146.6±101.1 mg/L; ferritin: 17245.4±5328.1 ng/mL). This is the smallest cluster considering the number of patients but with higher levels of CRP and ferritin. Finally, cluster 4 comprised 75 patients (age: 51.8±16.1 years; systemic score: 6.8±2.0; ESR: 93.6±23.6; CRP: 143.8±103.6 mg/L; ferritin: 2947.7±2818.6 ng/mL). Higher values of age and systemic score were recognised in this group of patients. No paediatric patients were included in this cluster.

### Clinical characterisation of derived clusters of patients with Still’s disease

All clusters were mainly characterised by fever, skin rash and joint involvement, as shown in table 1. However, cluster 4 was burdened by an increased rate of multiorgan involvement, and life-threatening complications; it appeared a more severe patient group than others. Specifically, a higher percentage of these patients showed sore throat (p=0.007), myalgia (p=0.027) and serositis, both pericarditis (p=0.006) and pleuritis (p<0.0001). Furthermore, these patients were more frequently characterised by multiorgan involvement, including lymph-node enlargement (p=0.007), splenomegaly (p=0.003) and liver disease (p<0.0001). In addition, cluster 4 was burdened by a higher rate of life-threatening complications, both MAS (p=0.008) and lung disease (p<0.0001). We did not have available lung biopsies to fully define this pulmonary involvement but the CT images were mainly reported to be attributed to the endogenous lipoid pneumonia/pulmonary alveolar proteinosis spectrum and inflammatory interstitial infiltration of the lung.

To better describe the derived disease clusters, ORs-like were exploited to evaluate how patient clinical features could be predictive of different cluster membership by multinomial logistic analysis, as summarised in table 2. Cluster 2 was identified as ‘base outcome’ in this evaluation. Patients included in cluster 1 were significantly characterised by skin rash (OR-like: 1.87; 95% CI: 1.04 to 3.38; p=0.037), myalgia (OR-like: 2.25; 95% CI: 1.25 to 4.05; p=0.007) and sore throat (OR-like:1.89; 95% CI: 1.07 to 3.34; p=0.027). Furthermore, an increased frequency of splenomegaly (OR-like: 1.94; 95% CI: 1.06 to 3.57; p=0.032) and MAS (OR-like: 3.67; 95% CI: 1.37 to 9.82; p=0.010) also distinguished these patients in cluster 1 when compared with those included in cluster 2. Assessing patients comprised in clusters 3, they were significantly burdened by an increased rate of MAS (OR-like: 4.65; 95% CI: 1.38 to 15.70; p=0.013) when compared with cluster 2. In addition, although not significant, a trend towards a negative association with male gender (OR-like: 0.42, 95% CI: 0.17 to 1.05; p=0.063) was observed in this cluster. Finally, patients within clusters 4 were significantly characterised by myalgia (OR-like: 2.56; 95% CI: 1.26 to 5.18; p=0.009), and sore throat (OR-like: 3.03; 95% CI: 1.49 to 6.15; p=0.002). Furthermore, an increased rate of liver involvement (OR-like: 2.29; 95% CI: 1.11 to 4.75; p=0.025), and occurrence of MAS (OR-like: 3.20; 95% CI: 1.08 to 9.48; p=0.036) were observed in these patients when compared with those comprised in cluster 2. Although not significant, patients comprised in cluster 4 showed a trend towards a less frequency of arthralgia (OR-like: 0.40; 95% CI: 0.15 to 1.03; p=0.058), whereas a higher rate of skin rash (OR-like: 2.06; 95% CI: 1.00 to 4.26; p=0.050). Furthermore, a higher frequency of lymph node involvement (OR-like: 1.95; 95% CI: 0.99 to 3.88; p=0.055) and a higher occurrence of lung disease (OR-like: 2.85; 95% CI: 0.85 to 9.54; p=0.088) were retrieved, even if not significant results.

**Table 2:** Multinomial logistic regression analysis exploiting ORs-like to evaluate patient clinical features as predictive factor of different cluster membership

	ORs-like	SE	95% CI	P value
Cluster 1: ‘Juvenile/Transitional’				
Male sex	0.59	0.17	0.33 to 1.04	0.067
Arthralgia	0.90	0.40	0.37 to 2.16	0.809
Arthritis	0.62	0.19	0.34 to 1.15	0.130
Skin rash	1.87	0.56	1.04 to 3.38	**0.037**
Sore throat	1.89	0.55	1.07 to 3.34	**0.027**
Myalgia	2.25	0.67	1.25 to 4.05	**0.007**
Lymph node involvement	1.61	0.46	0.92 to 2.84	0.096
Liver involvement	1.17	0.36	0.64 to 2.14	0.597
Spleen involvement	1.94	0.60	1.06 to 3.57	**0.032**
Pericarditis	1.03	0.48	0.41 to 2.59	0.943
Pleuritis	1.02	0.51	0.38 to 2.71	0.974
Abdominal pain	1.47	0.60	0.66 to 3.27	0.347
MAS	3.67	1.84	1.37 to 9.82	**0.010**
Lung disease	1.24	0.78	0.36 to 4.25	0.730
Cluster 2: ‘Uncomplicated’				
Base outcome				
Cluster 3: ‘Hyperferritinemic’				
Male sex	0.42	0.20	0.17 to 1.05	0.063
Arthralgia	0.56	0.35	0.17 to 1.89	0.352
Arthritis	0.57	0.27	0.23 to 1.44	0.237
Skin rash	1.51	0.70	0.61 to 3.72	0.373
Sore throat	1.51	0.67	0.63 to 3.61	0.354
Myalgia	1.35	0.60	0.56 to 3.23	0.507
Lymph node involvement	1.22	0.54	0.51 to 2.91	0.653
Liver involvement	2.21	1.04	0.88 to 5.54	0.090
Spleen involvement	0.71	0.35	0.27 to 1.85	0.481
Pericarditis	1.78	1.12	0.52 to 6.09	0.354
Pleuritis	1.32	0.87	0.36 to 4.82	0.677
Abdominal pain	0.83	0.59	0.21 to 3.30	0.796
MAS	4.65	2.89	1.38 to 15.70	**0.013**
Lung disease	1.03	0.96	0.17 to 6.40	0.971
Cluster 4: ‘Catastrophic’				
Male sex	0.99	0.34	0.51 to 1.92	0.966
Arthralgia	0.40	0.19	0.15 to 1.03	0.058
Arthritis	0.74	0.28	0.35 to 1.56	0.432
Skin rash	2.07	0.76	1.00 to 4.27	0.050
Sore throat	3.03	1.09	1.50 to 6.15	**0.002**
Myalgia	2.56	0.92	1.26 to 5.18	**0.009**
Lymph node involvement	1.96	0.68	0.99 to 3.88	0.055
Liver involvement	2.29	0.85	1.11 to 4.75	**0.025**
Spleen involvement	1.81	0.67	0.88 to 3.72	0.106
Pericarditis	1.57	0.78	0.59 to 4.14	0.364
Pleuritis	2.21	1.13	0.81 to 6.01	0.121
Abdominal pain	0.93	0.47	0.34 to 2.51	0.879
MAS	3.20	1.77	1.08 to 9.48	**0.036**
Lung disease	2.86	1.76	0.85 to 9.54	0.088
χ^2^ = 108.59; **p<0.0001**				

Bold values are statistically significant results.

MAS, macrophage activation syndrome.

### Disease patterns and mortality of derived clusters of patients with Still’s disease

In the subsequent follow-up, different disease courses and mortality rate were analysed according to derived patient clusters. Concerning mortality rate, we registered 19 Still’s disease-related death in this cohort, mainly due to multiorgan failure syndrome in the context of uncontrollable MAS and/or severe infections.

In cluster 1 and cluster 2, similar percentages of patients developed the three different disease patterns. A higher proportion of patients (58.1%) in cluster 3 experienced a monocyclic disease pattern, whereas a low percentage had a chronic disease course (12.9%). In cluster 4, a higher frequency of polycyclic disease course (40%) was observed. Comparing the rate of mortality, an increased rate of death (12%) was reported in patients in cluster 4 when compared with those in other clusters, as reported in table 1. In addition, as summarised in table 3, a multinomial model was exploited to evaluate the predictive strength of the derived clusters on the overall outcomes of patients. Cluster 4 resulted a significant predictor of mortality in these patients (OR-like: 11.02538; 95% CI: 2.19 to 55.60; p=0.004). After that, MAS was included in this multinomial model to evaluate its impact on outcomes of patients. Also in this case, cluster 4 resulted to be a significant predictor of mortality (OR-like 9.55;95% CI: 1.82 to 49.99; p=0.008). Interestingly, MAS showed to be a significant predictor of mortality of patients included in cluster 4 (OR-like: 5.99; 95% CI: 1.89 to 18.95; p=0.002). This finding was not observed in other derived clusters.

**Table 3:** Multinomial logistic regression analysis exploiting ORs-like to evaluate the predictive strength of the derived clusters on the overall outcomes of patients

	ORs-like	SE	95% CI	P value
Monocyclic pattern				
Base outcome				
Polycyclic pattern				
Cluster 1	Reference			
Cluster 2	1.42	0.43	0.78 to 2.57	0.252
Cluster 3	0.66	0.31	0.27 to 1.64	0.373
Cluster 4	1.99	0.72	0.98 to 4.04	0.058
Chronic pattern				
Cluster 1	Reference			
Cluster 2	1.31	0.44	0.68 to 2.53	0.424
Cluster 3	0.40	0.24	0.12 to 1.31	0.132
Cluster 4	1.45	0.60	0.65 to 3.26	0.366
Mortality				
Cluster 1	Reference			
Cluster 2	4.56	3.73	0.92 to 22.63	0.064
Cluster 3	Not estimable			
Cluster 4	11.03	9.10	2.19 to 55.60	**0.004**
χ^2^ = 21.68; **p=0.010**				
Monocyclic pattern				
Base outcome				
Polycyclic pattern				
Cluster 1	Reference			
Cluster 2	1.46	0.45	0.80 to 2.67	0.220
Cluster 3	0.65	0.30	0.26 to 1.62	0.358
Cluster 4	2.00	0.73	0.98 to 4.07	0.057
Male sex	0.96	0.25	0.58 to 1.60	0.884
MAS	1.25	0.47	0.60 to 2.61	0.552
Chronic pattern				
Cluster 1	Reference			
Cluster 2	1.28	0.44	0.66 to 2.51	0.468
Cluster 3	0.41	0.25	0.13 to 1.35	0.144
Cluster 4	1.36	0.57	0.60 to 3.07	0.461
Male sex	1.67	0.49	0.94 to 2.97	0.079
MAS	1.21	0.52	0.52 to 2.82	0.652
Mortality				
Cluster 1	Reference			
Cluster 2	5.99	5.07	0.94 to 31.48	0.064
Cluster 3	Not estimable			
Cluster 4	9.55	8.07	1.82 to 49.99	**0.008**
Male sex	2.36	1.29	0.80 to 6.91	0.118
MAS	5.99	3.52	1.89 to 18.95	**0.002**
χ^2^ = 37.48; **p=0.001**				

Bold values are statistically significant results.

MAS, macrophage activation syndrome.

## Discussion

Four distinct patient clusters in Still’s disease may be recognised by a clinical and laboratory multidimensional characterisation in a multicentre, observational, prospective study from GIRRCS AOSD-study group and AIDA Network Still Disease Registry. Although these clusters were similarly characterised by the typical triad of fever, arthritis and skin rash, some clinical differences may be observed. Cluster 4 was burdened by an increased rate of multiorgan manifestations and mortality, suggesting a more severe group of patients to be managed. Taking together these considerations, a clinically based stratification may be a meaningful approach, addressing the patient heterogeneity, reflecting possible differences in pathogenic mechanisms and variable therapeutic responses.

Based on a robust method for stratification, we derived and validated four patient clusters, with similar main clinical manifestations (ie, fever, joint involvement and skin rash), highlighting a disease continuum but also some clinical differences, specific for each cluster, accounting for the patient heterogeneity. Our stratification approach was focused on identifying, characterising and validating patient clusters, considering that any clinical phenotype may likely be underpinned by diverse networks of dysregulated pathogenic pathways, at least partially.^34^ In fact, the resulting patient heterogeneity may be presumably driven by differences in underlying molecular pathology also resulting in variable responses to therapies.^34 35^ Thus, the identification of our four patient clusters may possibly overcome those major obstacles which strongly limited the development of more and more effective therapeutic strategies for patients with Still’s disease. In fact, it possible that different clusters could advocate a diverse therapeutic management to improve the outcomes of patients over time and increasing the possibility to reach a drug free-remission. In addition, our findings may support the use of these derived clusters in dissecting the biological differences of the disease, informing clinical management and improving the design of future studies. Due to the rarity of the disease making difficult to arrange any prospective study, these data may be important in reducing the number of required patients to obtain clinically relevant results. According to the principles of precision medicine, our results may also help in optimally tailoring the management to the appropriate patient populations.^36^ In fact, based on this proposed patient profiling, clinicians could balance appropriate escalation of therapy, minimising the exposure to iatrogenic side effect and avoiding unnecessary costs, in those patients with a less severe disease. In parallel, the recognition of more severe patients could guide the clinicians when to apply additional resources. Taking together all these observations, cluster analysis may provide useful and relevant insights to dissect the complex clinical heterogeneity in Still’s disease to improve the management of these patients.

Different from previous preliminary derivation study of patient clusters,^22^ a more rigorous methodological procedure was employed in this evaluation. First, a specific sample size was adequately calculated to establish how many clusters could be present within the collected data, and to what extent the cluster membership of individual observations could be accurately classified. The k-means algorithm with Euclidean metric was performed, setting 100 random assignments to the cluster seeds, preventing possible dependence of clusters from the choice of points at the onset. In addition, z-scores of cluster variables were derived to account for the different units of the selected variables. In the present assessment, these findings were also based on a prospective study which was built accordingly, and not on a retrospective evaluation as the preliminary derivation.^22^ Finally, in the present study, regression analyses were exploited in accurately describing the clinical characteristics of patients according to different clusters.

In this analysis, any derived cluster was compared with each other to define both specific clinical manifestations and disease outcomes. In cluster 1, the youngest patients were the majority displaying lower values of CRP and higher levels of systemic score than cluster 2 and cluster 3. Clinically, these patients were also characterised by sore throat, myalgia and splenomegaly. The high rate of sore throat may appear as a possible discrepancy with previous literature considering lower age of onset in these patients.^7 8^ However, we assessed a majority of adult patients, and the low percentage of sore throat in paediatric patients could be mainly attributed to the lack of recognition rather than a disease difference. Sore throat is usually less reported by young kids. Furthermore, this clinical characteristic is required by Yamaguchi criteria,^24^ whereas it is not present in ILAR criteria.^27^ Although MAS was observed in 20% of patients, a low rate of mortality was shown, confirming that older age is one of the major determinants of poor prognosis during this life-threatening complication.^37 38^ Due to these characteristics, we named this cluster as ‘Juvenile/Transitional’.

Assessing cluster 2, this was the largest derived patient group. These patients were identified by the lowest levels of ESR, ferritin and systemic score. Only a minority of these patients presented life-threatening complications. Thus, cluster 2 appeared to include those patients more frequently attending the rheumatologic outpatient clinics. Together with the classical triad of symptoms, these patients showed a fivefold increase of ferritin (around 1000–1500 ng/mL), outlining the most common and less severe clinical phenotype of patients with Still’s disease. For these reasons, we called this cluster as ‘Uncomplicated’.

Patients comprised in cluster 3 were identified by the highest levels of CRP and ferritin, the latter showing even a 40-fold increase than normal values. Lower levels of systemic score were also observed in this cluster than cluster 1 and cluster 4. These patients showed a less pronounced multiorgan involvement. A frequency of MAS around 20% was retrieved in these patients confirming the close relationship between increased levels of ferritin and occurrence of this complication.^39 40^ Patients in this cluster were mainly characterised by a monocyclic pattern of the disease. Despite the highest of ferritin, we may speculate that the lack of systemic multiorgan involvement may lead to a more favourable outcome than those observed in other clusters. In fact, hyperferritinemia may discriminate the occurrence of MAS,^41^ but the mortality of these patients may be better predicted by additional features such as the multiorgan manifestations and high levels of CRP.^9 28 37 41^ In addition, the highest proinflammatory markers observed in these patients in cluster 3 may probably facilitate an early diagnosis and a timely treatment thus improving the outcomes. This cluster could be named as ‘Hyperferritinemic’.

Finally, cluster 4 was derived according to the highest values of age and systemic score. Levels of ferritin were lower in respect to cluster 3 but higher than cluster 1 and cluster 2. These patients were characterised by myalgia, sore throat, serositis and liver involvement. Furthermore, a rate of MAS around 20% was shown in association with lung involvement. The latter has been recently suggested to excessively amplify the immune response, contributing to a massive release of proinflammatory mediators, inducing the occurrence of MAS, and leading to a poor outcome.^10 30 31 42^ Therefore, patients in this cluster 4 could be affected by the cytokine storm syndrome, defined as the concomitant presence of a hyperinflammatory process and multiorgan manifestations.^43 44^ These data probably explain the highest mortality in this cluster when compared with other clusters and confirming a difficult to treat clinical phenotype. In fact, cluster 4 resulted to be a predictive factor for mortality, mainly in patients with MAS. Supplementary features in adults may influence the prognosis in explaining the poor outcomes of these patients and complicating their management, such as smoking habit, comorbidities and ageing related frailty.^37 38 45–47^ The latter is a syndrome characterised by a decrease of strength, reduced physiological function and increased individual vulnerability, generally associated with older age.^38^ According to these features, this cluster may be termed as ‘Catastrophic’.

Taking together all these findings, the basis of a new taxonomy may be laid in Still’s disease. This is of importance in improving the management of these patients. In fact, creating a new taxonomy of disease clusters may identify possible different aetiologies of the disorder to be fully understood, and consequent better therapeutic strategies may be devised and tested accordingly. The relevance of these issues has been recently highlighted by Paediatric and Adult Rheumatologists in the context of a EULAR/PRES initiative to produce recommendations for the diagnosis and management of patients with Still’s disease (EULAR ongoing initiative QoC011- https://www.eular.org/ongoing-initiatives).

We are aware about some limitations of this study. Although clustering analysis is a useful tool, the method is sensitive to the choice of clustering method and assessment metrics, and there is still no consensus on the optimal approach.^18^ A further limitation is that GIRRCS AOSD-study group and AIDA Network Still Disease Registry were not originally designed with stratification as the primary objective, although we combined two large cohorts and only patients with a prospective follow-up were assessed. Nevertheless, multicentre studies have some disadvantages regarding the difference in clinical practice among centres, which may be a confounding factor for the interpretation and homogenisation of the results. However, the strength of our multicentre study derives from providing a ‘real-life’ evaluation of the clinical heterogeneity of patients with Still’s disease. However, a minority of our patients had a paediatric onset of Still’s disease, suggesting the need of further studies to fully confirm our results in this subset. In addition, mainly Italian centres were included in this evaluation, possibly limiting the generalisation of our findings to populations from different geographic areas of origin. Finally, our study was not specifically designed to assess the influence of therapeutic strategies on disease outcomes. However, despite diverse clinical manifestations, patients showed a similar treatment pattern. Thus, further studies are advocated in better tailoring the therapeutic strategies on the clinical picture to improve the long-term outcomes of these patients and the achievement of drug-free remission.

In conclusion, four distinct patient clusters in Still’s disease may be recognised by a clinical and laboratory multidimensional characterisation (ie, ‘Juvenile/Transitional’, ‘Uncomplicated’, ‘Hyperferritinemic’ and ‘Catastrophic’). Each one of these has some different clinical features from others. Cluster 4 was burdened by an increased rate of multiorgan manifestations and mortality, proposing a more severe patient group to be managed. Finally, our findings may suggest that clinical picture-based stratification may be a robust and clinically meaningful approach, addressing the heterogeneity of patient picture and reflecting differences in pathogenetic mechanisms, possibly explaining the variability of the therapeutic responses. On these bases, future-specific designed studies may be arranged considering different disease clusters to fully clarify these issues and to improve the management of patients with Still’s disease.

## Data Availability

All data relevant to the study are included in the article or uploaded as supplementary information.
