# Supplementary material for: Derivation and validation of four patient clusters in Still’s disease, results from GIRRCS AOSD-study group and AIDA Network Still Disease Registry
Source: RMD Open. 2023 Nov 20;9(4):e003419. doi: 10.1136/rmdopen-2023-003419 (PMC10660445; doi:10.1136/rmdopen-2023-003419)

Supplementary Table 1. Cluster means standardized according to z-scores.

Clusters	Age	Systemic score	ESR	CRP	Ferritin
1	-0.67898749	0.20836320	0.6611048	-0.4520566	-0.25034722
2	0.13809507	-0.54304646	-0.8867966	-0.3264653	-0.36492329
3	-0.02664983	-0.07922267	-0.1437485	0.9086280	2.67651075
4	0.81644717	0.64005443	0.5591883	0.8747547	-0.09962296

Abbreviations. ESR: erythrocyte sedimentation rate, CRP: C reactive protein

Supplementary Figure 1. Elbow plot and visualization of Clusters

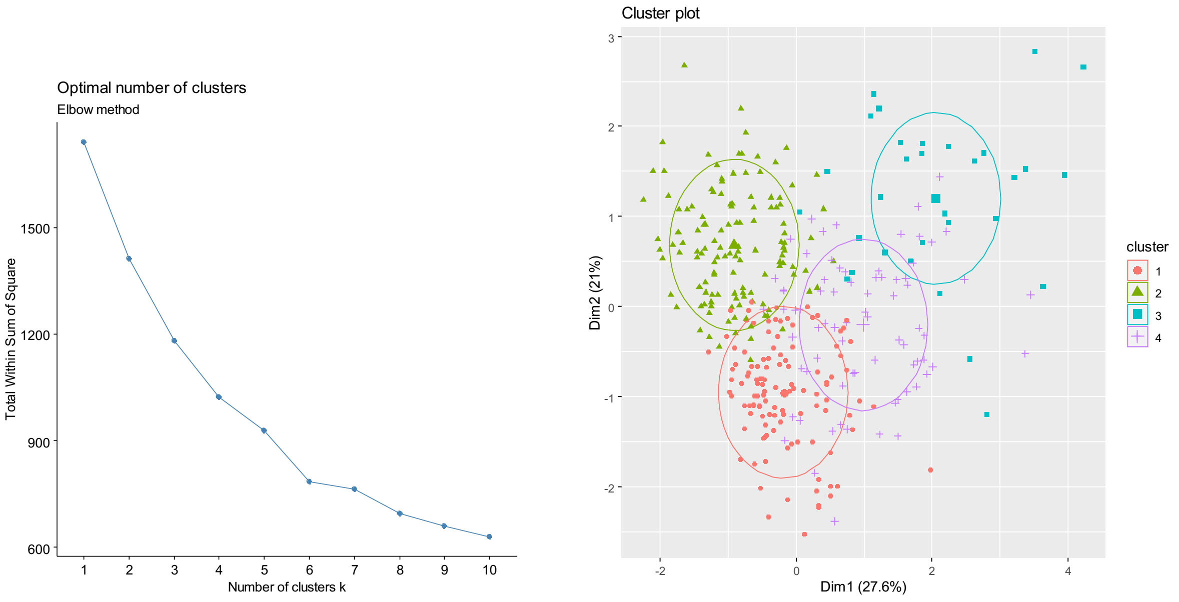

Supplement: Supplementary data [file rmdopen-2023-003419supp001.pdf]
